# Namib Desert dune/interdune transects exhibit habitat-specific edaphic bacterial communities

**DOI:** 10.3389/fmicb.2015.00845

**Published:** 2015-09-04

**Authors:** Sandra Ronca, Jean-Baptiste Ramond, Brian E. Jones, Mary Seely, Don A. Cowan

**Affiliations:** ^1^Department of Genetics, Centre for Microbial Ecology and Genomics, University of PretoriaPretoria, South Africa; ^2^DuPont Industrial BiosciencesLeiden, Netherlands; ^3^Gobabeb Research and Training CentreWalvis Bay, Namibia; ^4^School of Animal, Plant and Environmental Sciences, University of the WitwatersrandJohannesburg, South Africa

**Keywords:** deterministic assembly, open soil, habitat filtration, dune transects, environmental gradient, arid ecosystems

## Abstract

The sand dunes and inter-dune zones of the hyper-arid central Namib Desert represent heterogeneous soil habitats. As little is known about their indigenous edaphic bacterial communities, we aimed to evaluate their diversity and factors of assembly and hypothesized that soil physicochemistry gradients would strongly shape dune/interdune communities. We sampled a total of 125 samples from 5 parallel dune/interdune transects and characterized 21 physico-chemical edaphic parameters coupled with 16S rRNA gene bacterial community fingerprinting using T-RFLP and 454 pyrosequencing. Multivariate analyses of T-RFLP data showed significantly different bacterial communities, related to physico-chemical gradients, in four distinct dune habitats: the dune top, slope, base and interdune zones. Pyrosequencing of 16S rRNA gene amplicon sets showed that each dune zone presented a unique phylogenetic profile, suggesting a high degree of environmental selection. The combined results strongly infer that habitat filtering is an important factor shaping Namib Desert dune bacterial communities, with habitat stability, soil texture and mineral and nutrient contents being the main environmental drivers of bacterial community structures.

## Introduction

Despite extreme surface conditions, notably (hyper-) aridity, wide daily temperature fluctuations, high UV radiation and oligotrophy, edaphic microbial communities have been shown to flourish in desert soils (Makhalanyane et al., 2015). The Namib Desert of south-western Africa is considered to be one of the oldest desert regions on Earth (Eckardt and Spiro, 1999). It is characterized by a wide range of different soil environments including gravel plains, sand dunes, inselbergs, escarpments, river beds, salt pans and playas (Seely, 2012; Eckardt et al., 2013a). In the central Namib Desert, studies have notably determined that local soil physicochemical conditions and climate played a significant role in the assembly of edaphic and hypolithic communities were (e.g., Stomeo et al., 2013; Warren-Rhodes et al., 2013; Ramond et al., 2014; Gombeer et al., 2015).

However, and despite representing over 41% of the total desert land surface (Seely, 2012), the detailed microbial ecology (as determined by modern molecular tools) of the Namib Sand Sea has to date poorly been assessed and essentially focused on fungal communities (Jacobson, 1997; Jacobson and Jacobson, 1998; Jacobson et al., 2015). The dispersal and colonization of mycorrhizal fungal communities were shown to depend on sand stability and moisture availability, respectively (Jacobson, 1997). And, moisture-activated decomposing fungi were observed on the litter of the perennial Namib dune grass *Stipagrostis sabulicola* and in dune sands (Jacobson and Jacobson, 1998; Jacobson et al., 2015). Recently, a T-RFLP fingerprinting-based study compared the edaphic bacterial community assemblages from multiple soil surface geologies and lithologies of the central Namib Desert. It showed that (i) interdune bacterial communities were significantly different than those of the gravel plains and riverbeds and that (ii) the soil physicochemistry and lithology of the different interdunes sampled were important in structuring their indigenous communities (Gombeer et al., 2015).

Dune morphology and dynamics have in contrast extensively been studied in the Namib Desert. Three geographically distinct dune morphology patterns have been characterized: transverse dunes to the west (i.e., toward the coast), linear and complex linear dunes in the center and star dunes in the east (i.e., inland) (Livingstone, 2013). Similarly, an east/west sand color gradient, from yellowish brown (west) to much redder (east), has been described and attributed to variations in iron oxide and clay mineral concentrations (Walden and White, 1997; Livingstone, 2013). Within dune slopes, a complex moisture gradient has been shown to influence the number of species perennial grasses as well as their growth strategies and distribution (Yeaton, 1988; Seely, 1990). Dunes have also been described to be highly dynamic environments, as they are constantly modified by surface shear forces and mechanical stresses. For example, it has been reported annual transverse dune crest migrations varying from 4 to 56 m (Livingstone, 2013). Wind has also been shown to alter the top of wind-exposed slopes (or stoss slopes) and sand accumulation to dominate along lee slopes (or downwind slopes) (Lancaster, 1985; Eckardt et al., 2013a). The interactions between wind erosion and sediment deposition lead to discernible patterns in grain size and sorting gradients both on individual dune slopes and between dune and interdune areas.

In such a dynamic and heterogeneous habitat, dune microbial communities have thus to cope with numerous stresses, including environmental physical instability, fluctuating soil physico-chemical properties, low water availability, high temperatures, oligotrophy and alkaline pH (typically around 8) (Makhalanyane et al., 2015); all of which have been shown to affect both community diversity and function (Andrew et al., 2012; Kuske et al., 2012; Yu and Steinberger, 2012a; Makhalanyane et al., 2015).

Using the Kahani dune system in the Namib Sand Sea as a model dune environment, we aimed to determine drivers shaping edaphic bacterial community diversity in four dune biotopes (dune top, slope, base, and interdune). We hypothesized that in such a dynamic environment, strong soil physico-chemical gradients would structure bacterial communities; i.e., that deterministic processes would lead to dune habitat-specific communities. Our related null hypothesis stated that high dispersal rates, due to the important dynamicity of dune ecosystems, would lead to homogenized communities (Leibold and Norberg, 2004). To test these hypotheses, we collected 125 soil samples from 5 parallel dune/interdune transects in a single Namib Desert dune system; i.e., from the summit of a western dune to the crest of an eastern dune. For each sample, we recorded 21 edaphic physico-chemical parameters and characterized the bacterial community structure by 16S rRNA gene T-RFLP analyses. We also performed 16S rRNA gene pyrosequencing on representative samples of 7 putative dune habitats found across the dune/interdune transect, namely the east and west dune tops, slopes bases and the interdune, to investigate their taxonomic composition and assess if they presented different assemblages.

## Materials and methods

### Study site and soil sample collection

**Figure 1:**
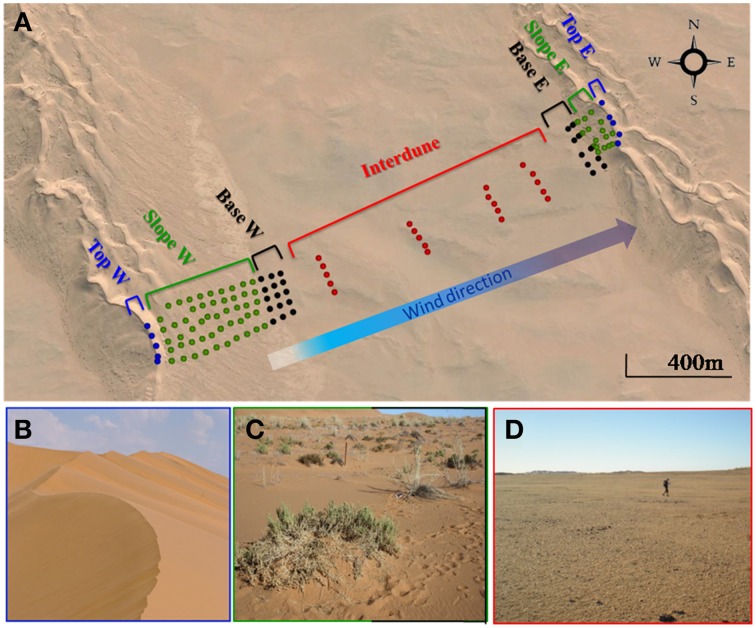
**The Kahani dune system sampled in the Namib Desert. (A)** Google Maps representation of the sampling site showing the individual soil samples collected. Filled circles represent the 125 soil samples from the 5 dune/interdune transects, from dune Tops (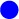), Slopes (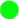), Bases (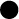) or Interdune (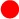). E, East; W, West. **(B)** Photograph of the dune Top (west) devoid of vegetation. **(C)** Photograph of the dune Slope (W) with sparse perennial grasses (*Stipagrostis sabulicola* and *Trianthema hereroensis*). **(D)** Photograph of the barren Interdune.

Soil samples were collected in April 2013 in the Kahani dune system (S 23°35.830′/E 15°01.800′), a typical complex linear dune in the Namib Desert Sand Sea (Figure 1). 25 soil samples were collected from each of five parallel (40 m apart) 2 km long dune/interdune linear transects from the west (stoss slope) to the opposite east dune (lee slope) resulting in a total of 125 soil samples (Figure 1). Each transect was divided into 4 morphologically distinct zones: dune Top, Slope, Base and Interdune. Sampling points were regularly distributed; every 40 m from the west dune Top to its base, every 200 m in the Interdune area and every 20 m on the eastern dune up to the dune Top, as the west facing dune was typically shorter and steeper than the east facing dune (Figure 1). Surface soil samples (0-5 cm depth) were collected (avoiding grasses on the dune Slope to minimize any rhizosphere bias), and each individual sample comprised the pooling of four pseudo-replicate soils in a 1 m^2^ area. Samples were stored at 4°C for soil chemistry analysis, at −20°C for molecular analysis and processed immediately for fluorescein di-acetate (FDA) assays.

### Soil physico-chemistry analyses

All analyses were performed at the Soil Science Laboratory of the University of Pretoria. Prior to analyses of 21 edaphic parameters, the soil samples were sieved (2 mm mesh) and dried overnight at 37°C. pH was measured in 10 g soil slurries (1:2.5 soil/deionized water) with a pH meter (Crison basic 20, Barcelona, Spain) (Eckert and Sims, 1995). Soil nitrate (NO^−^_3_-N) and ammonium (NH^+^_4_-N) were determined by extraction (2M KCl) and steam distillation with subsequent titration performed as described by Keeney and Nelson (1982). Soil total carbon percentage (C) was measured by oxidizing the organic material with chromic acid and titrating the excess dichromate (Walkley, 1935; Nelson, 1982). Cation exchange capacity (CEC) was determined using an ammonium acetate solution as extractant for the exchangeable and water-soluble cations with steam distillation to separate the ammonia (Rhoades, 1982). Ammonium acetate extraction was also used to measure salt concentrations (Na, K, Mg, and Ca) using inductively coupled plasma atomic emission spectroscopy (ICP-OES) (Spectro Genesis, Spectro Analytical Instruments GmbH, Germany). The Bray-1 method (Bray and Kurtz, 1945) was used to quantify extractable phosphorous (P). Metals (Fe, Al, and Mn) were extracted from 10 g of soil using EDTA buffer and quantified by ICP-OES after filtration through a 0.45 μm Millipore filter (EMD Millipore Corporation, Billerica, MA, USA).

Particle size distributions were determined using the ASTM protocol D422-63 on 50 g of soil sample (ASTM, 1985). The hydrometer method (Bouyoucos, 1962) was used to measure the size distribution of the silt and clay fractions obtained from the ASTM protocol. Granulometry analysis contributed to the formation of 8 variables: very coarse sand (VCS), coarse sand (CS), medium sand (MS), fine sand (FS), very fine sand (VFS) and the portion of the silt plus clay (SC), silt (ST), and clay (CY).

### Fluorescein di-acetate degradation assay

FDA hydrolysis, as an indicator of general bacterial metabolic activity, was measured using the protocol of Green et al. (2006). 0.5 g of soil was mixed with 12.5 mL of sterile *phosphate buffered* saline (PBS, pH 7.4) and 0.25 mL of 4.9 mM FDA dissolved in acetone and subsequently incubated at 28°C for 1 h under constant agitation. FDA hydrolysis was stopped by mixing 40 μL of acetone with 1 mL of soil slurry. After centrifugation (8800 × g, 5 min.) fluorescence was measured with a portable fluorometer (Quantifluor, Promega, Madison, USA), calibrated against a standard curve. FDA activity was expressed as μg fluorescein per g soil per hour (μg fluorescein g^−1^ soil h^−1^).

### Metagenomic DNA extraction, PCR amplification and T-RFLP

Metagenomic DNA was extracted from 0.5 g of soil using the PowerSoil™ DNA Isolation Kit (MoBio, West Carlsbad, CA, USA) following the manufacturer's instructions.

PCR reactions were carried out in a Thermo Cycler (BioRad) in 50 μL final volume reactions containing 1X PCR Buffer A, 1X Enhancer, 0.2 mM each dNTP, 0.5 μM of each primer (E9F: 5′-GAGTTTGATCCTGGCTCAG-3′/U1510R: 5′-GGTTACCTTGTTACGACTT-3′) (Reysenbach and Pace, 1995; Hansen et al., 1998), 4% DMSO, 0.5 U of KAPA2G Robust DNA polymerase (KAPA, USA) and 10 ng of template DNA. Thermal cycling conditions were as follows: 3 min denaturation at 95°C followed by 30 cycles with denaturation at 95°C for 30 s, annealing at 55°C for 30 s, and elongation at 72°C for 90 s with a final elongation at 72°C for 10 min. This reaction yielded 1.5 kb amplicons.

For T-RFLP analysis, the forward E9F primer was FAM-labeled and the PCR products were purified using the NucleoSpin Kit™ (Macherey-Nagel, Germany) and digested overnight using *Hae*III (Fermentas, USA). After a second purification, electrophoretic separation of the terminal-restriction fragments (T-RFs) was performed using an ABI3130XL sequencer (Applied Biosystems, USA). The retrieved T-RFLP profiles were analyzed using Peak Scanner 1.0 (Applied Biosystems; http://www.appliedbiosystems.com). True peaks and fragments of similar size were identified and binned using the software R and Perl (Abdo et al., 2006). The term OTU (Operational Taxonomic Unit) is used to refer to individual terminal restriction fragments (T-RF) in T-RFLP patterns, with recognition that each OTU may comprise more than one distinct bacterial ribotype.

### Statistical analyses

Principal Component Analyses (PCA) was performed (*prcomp R* function) on standardized soil chemistry variables and granulometric parameters (Oksanen, 2013). T-RFLP data were Hellinger-transformed (Legendre and Gallagher, 2001) and used to calculate Bray-Curtis dissimilarity matrices, which were further visualized using 3D non-metric multidimensional scaling (3D-nMDS) (Primer 6 software; Primer-E Ltd, UK). Permutational multivariate analysis of variance (PERMANOVA), function *Adonis* (vegan package for R), was used to test for significant differences between sample groups along the dune/interdune transects. Distance based redundancy analysis (dbRDA) was used to determine correlations between bacterial community structures and habitat parameters (e.g., soil chemistry and granulometry) (*vegan* package for R). Negative eigenvalues were transformed by taking the square root of dissimilarities.

An analysis of multivariate homogeneity of group dispersions (variances) (*betadisper* function in vegan package for R) was used to test if one or more of the dune zones was significantly more variable than the others. The test was performed on standardized soil physico-chemical properties and bacterial community structures (relative abundance of OTUs).

Edaphic variables significantly correlated, or “redundant” (based on PCA analysis), were grouped for dbRDA analysis in order to decrease co-linearity and noise (Table 1) (Legendre and Legendre, 2012), yielding 15 independent variables: pH, NO^−^_3_-N, NH^+^_4_-N, C, CEC, Na, K, P, Mg (also representing Ca), Fe (also representing Al and Mn), very coarse sand (VCS), coarse sand (CS), medium sand (MS), fine sand (FS) and very fine sand (VFS) (representing silt plus clay [SC], silt [ST] and clay [CY]). Tukey's HSD (honest significant difference) test was used to test for differences in averaged bacterial activity between dune areas.

**Table 1:** **Soil physico-chemistry of the four dune/interdune soil types studied (Top, Slope, Base and Interdune)**.

**Variable**	**Code**	**RDA group**	**Top**	**Slope**	**Base**	**Interdune**
**SOIL CHEMISTRY**						
Phosphorus (mg kg^−1^)	P	**P**	6.54 ± 0.69	9.13 ± 0.30	17.84 ± 0.99	30.04 ± 1.11
Organic carbon (%)	C	C	0.04 ± 0.01	0.04 ± 0	0.05 ± 0.01	0.07 ± 0.001
Ammonium (NH^+^_4_-N) (μg g^−1^)	NH4	NH4	8.74 ± 0.66	8.23 ± 0.19	8.58 ± 0.17	8.67 ± 0.18
Nitrate (NO^−^_3_-N) (μg g^−1^)	NO3	NO3	6.52 ± 0.43	6.87 ± 0.15	6.79 ± 0.24	6.93 ± 0.24
Potassium (mg kg^−1^)	K	K	2180.30 ± 44.07	1444.51 ± 50.49	2175.13 ± 75.31	2625.23 ± 36.59
Magnesium (mg kg^−1^)	Mg	**Mg**	317.54 ± 9.01	267.20 ± 10.65	581.60 ± 30.52	856.23 ± 18.94
Calcium (mg kg^−1^)	Ca	**Mg**	4914.84 ± 220.18	4725.15 ± 160.22	9375.07 ± 486.57	13702.48 ± 470.59
Sodium (mg kg^−1^)	Na	Na	1634.07 ± 49.26	965.52 ± 29.39	718.06 ± 31.19	508.66 ± 19.96
pH	pH	pH	8.59 ± 0.47	8.30 ± 0.11	7.82 ± 0.20	7.75 ± 0.26
Cation exchange capacity (cmol^+^ kg^−1^)	CEC	CEC	5.31 ± 0.58	5.11 ± 0.11	5.69 ± 0.16	6.40 ± 0.22
Aluminium (mg kg^−1^)	Al	**Fe**	22.34 ± 1.08	21.07 ± 0.59	35.59 ± 2.23	56.93 ± 2.47
Iron (mg kg^−1^)	Fe	**Fe**	8.19 ± 0.24	7.12 ± 0.14	10.30 ± 0.6	14.46 ± 0.91
Manganese (mg kg^−1^)	Mn	**Fe**	3.10 ± 0.03	3.36 ± 0.05	5.24 ± 0.25	8.22 ± 0.28
**GRANULOMETRY**						
Very course sand (%)	VCS	VCS	0.05 ± 0.04	0.34 ± 0.08	0.63 ± 0.09	0.58 ± 0.15
Coarse sand (%)	CS	CS	0.61 ± 0.84	6.43 ± 0.7	4.64 ± 0.83	1.30 ± 0.12
Medium sand (%)	MS	MS	26.56 ± 7.42	57.56 ± 1.43	36.14 ± 2.78	16.62 ± 1.20
Fine sand (%)	FS	FS	70.60 ± 7.32	31.12 ± 1.46	44.02 ± 2.22	44.31 ± 1.26
Very fine sand (%)	VFS	**VFS**	1.01 ± 0.28	3.83 ± 0.31	11.53 ± 1.44	29.24 ± 0.98
Silt & Clay (%)	SC	**VFS**	0.01 ± 0.01	0.09 ± 0.01	1.39 ± 0.26	4.57 ± 0.29
Silt (%)	ST	**VFS**	0.00	0.05 ± 0.03	0.13 ± 0.06	0.77 ± 0.15
Clay (%)	CY	**VFS**	0.00	0.01 ± 0.01	0.76 ± 0.1	1 ± 0

*All values are given as the mean of ± standard error. In bold are indicated the variables grouped for the RDA analysis presented in **Figure 7***.

#### 454 Pyrosequencing

Extracted metagenomic DNA samples from 7 dune zones (Tops E+W, Slopes E+W, Bases E+W and Interdune) were pooled. Bacterial 16S rRNA gene amplicons were generated with the primer set 27Fmod (5′-AGRGTTTGATCMTGGCTCAG-3′)/519Rmodbio (5′-GTNTTACNGCGGCKGCTG-3′) and sequenced using a Roche 454 FLX titanium next-generation sequencer by Mr DNA Laboratories (Shallowater, Texas, USA). The sequences are available at the NCBI Sequence Read Archive under the accession number SRP059482.

#### Pyrosequencing data analyses

Analyses were carried out using the mothur software package (Schloss et al., 2009). Sequences with ambiguity, and/or with homopolymeric stretches longer than 8 bp, or shorter than 200 bp were removed with no barcode and primer mismatches accepted. This resulted in reads with sizes ranging from 232 to 314 bp. Chimeras were removed using Perseus (Quince et al., 2011). Sequences were aligned to the reference SILVA database and clustered into operational taxonomic units (OTUs) at the species (97% similarity) level using the average neighbor settings in mothur. Each OTU was assigned a taxonomic classification with reference to the Ribosomal Database Project (RDP; http://rdp.cme.msu.edu/index.jsp). Collector's curves were produced using the Chao1 diversity index and the coverage using Good's coverage estimator. The community composition of the sand dune zones was compared with a UPGMA tree based on the Jaccard index. Indicator species analysis was conducted using the *multipat* function of the indicspecies package in R.

## Results

### Dune soil physico-chemistries

**Figure 2:**
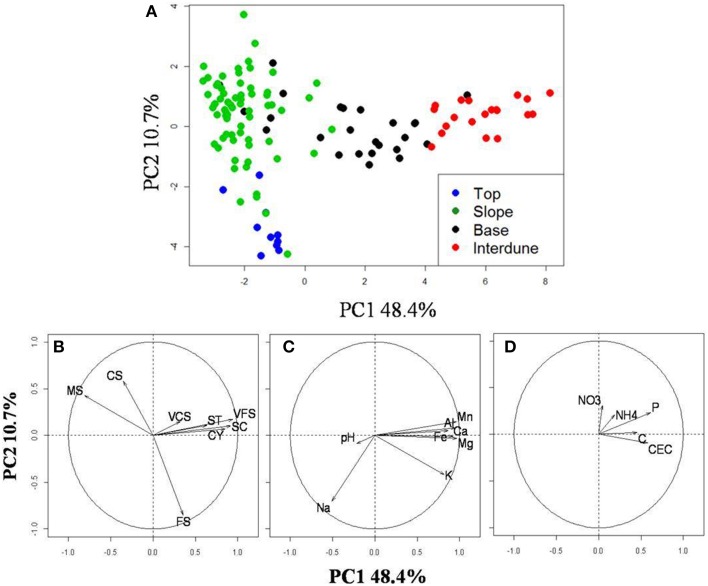
**Analysis of the dune edaphic physico-chemical parameters. (A)** Principal component analysis (PCA) on 21 standardized edaphic variables from the 125 soil samples collected from the dune Tops (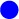), Slopes (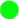), Bases (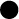), or the Interdune (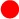). Vectors indicate the strength (length) and direction (arrow orientation) of single variable gradient. **(B)** Soil granulometry gradients; **(C)** Metals, minerals and pH and **(D)** the other soil properties measured. The code corresponding to each variable is described in Table 1.

Principal Component Analysis of 21 physico-chemical parameters from all 125 independent soil samples (Figure 2A) along the five dune-interdune transects (Figure 1) shows Top and Slope samples clearly separated along the PC2 axis (Figure 2A), explaining 10.7% of the sample variation. Dune Base samples cluster between Slope and Interdune samples (Figure 2A). Top and Slope samples clearly separate from those of the Interdune along PC1, which explains the majority of physico-chemical soil variation (48.4%). The most significant result to emerge from our analysis is the observation that each of the four sites examined, namely [dune] Top, Slope, Base and Interdune, was distinct and significantly different (*adonis R*^2^ = 0.477, *p* < 0.001) with respect to soil physico-chemistry. The test for multivariate homogeneity of group dispersions (*betadisper*) showed that variations in soil physico-chemical properties within dune zones were not significantly different (*p* = 0.14). No statistically significant differences in physico-chemical soil properties were found between dunes slopes exposed to different wind regimes (i.e., the lee or stoss slopes) (data not shown).

Soil texture analysis (Figure 2B) also shows separation of the four dune zones with an important fraction of fine sand in the Top zone and proportionally more coarse and medium sands in the Slope and Base areas (Table 1). The Interdune was characterized by a higher proportion of very fine sand, silt and clay than the other zones of the dune (Table 1). Dune Top samples had an elevated Na content and a higher pH (Table 1). Concentrations of minerals (K, Ca and Mg) and metals (Fe, Al) were lowest on dune Slopes (Figure 2C) and highest in the Interdune samples (Table 1) and inversely correlated with pH (Figure 2C). All other edaphic properties measured, including C, P, pH, CEC, NO^−^_3_-N, NH^+^_4_-N, and VCS (Table 1), were only very weakly correlated with the PC1 axis (Figure 2D) and do not appear to contribute to the dune/interdune physico-chemical gradients. Data for the dune Base (Table 1) was generally intermediate between Slope and Interdune. To conclude, this comprehensive dune soil physico-chemistry analyses clearly defined four dune biotopes; i.e., Top, Slope, Base and Interdune.

### Dune zone-specific bacterial community structure and diversity

**Figure 3:**
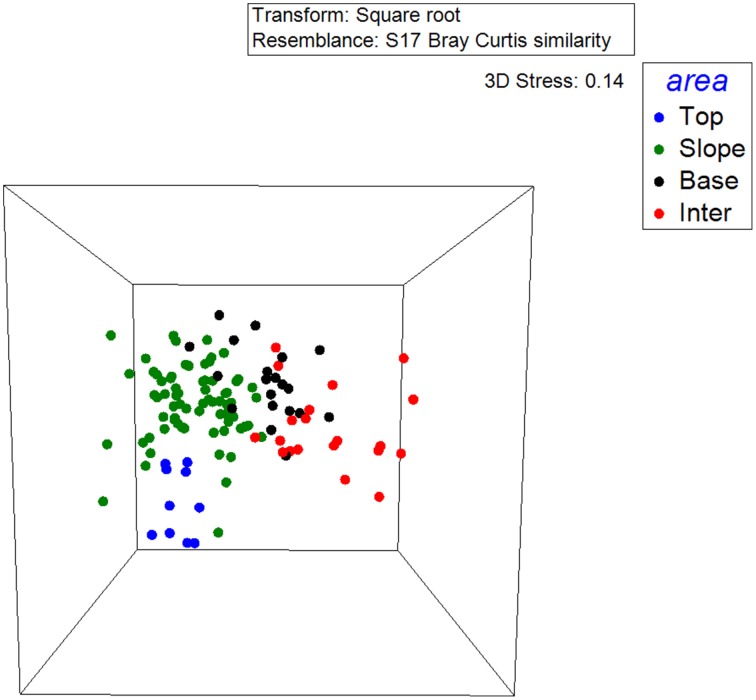
**Three-dimensional non-metric multidimensional scaling (3D-NMDS) plot of Bray-Curtis similarity matrixes of Namib Desert dune bacterial community structures determined by 16S rRNA genes T-RFLP**. Points represent the composition of a community in 3D multidimensional space, and the distance between any two points represents the difference between those two communities. The good quality of the ordination is indicated by a low stress value (3D stress = 0.14).

16S rRNA gene T-RFLP analysis yielded between 12 and 105 OTUs per individual sample (α-diversity) for each of the 125 samples. The *betadisper* function for multivariate homogeneity indicated that variances in the relative abundance of OTUs within dune zones were not significant (*p* = 0.59). Dune Tops showed the lowest bacterial diversity (41 OTUs), while the Slope was the most diverse (80 OTUs) followed by the Base and Interdune with 62 and 53 OTUs, respectively. Only 16 OTUs (15%) were shared by the bacterial communities of the four dune biotopes (Figure S1A), with samples from the Slope showing the highest number of unique OTUs (16). The 3D-NMDS plot showed that dune bacterial communities cluster according to their soil biotope of origin; i.e., Top, Slope, Base and Interdune (Figure 3). This was confirmed by PERMANOVA analysis (*adonis R*^2^ = 0.31, *p* = 0.001) and consistent with the analysis of soil physico-chemistry (Figure 2A).

Pyrosequencing analysis of 16S rRNA gene PCR amplicons yielded a total of 5204 OTUs (defined at 97% sequence similarity, Table 2; Figure S2B), of which 2741 (47.3%) were singletons. Dune Tops (E+W) showed the highest number of unique OTUs (1154) followed by the dune Bases (1170), Slopes (971), and the Interdune (775) samples. Only 88 OTUs (2%) were shared between the four dune biotopes, which is broadly consistent with the T-RFLP analysis (Figures S1A,B).

**Table 2:** **Distribution and diversity of OTUs (97% cut-off) in the seven dune zones studied**.

**Dune zone**	**Reads**	**Coverage**	**Observed OTUs**	**Chao1**
Top E	6899	0.965	782	983.71
Top W	11,709	0.964	1193	1663.27
Slope E	7089	0.939	884	1461.33
Slope W	9669	0.933	1307	2234.56
Base E	7671	0.907	1329	2507.43
Base W	6836	0.905	1209	2289.56
Interdune	4653	0.836	1251	2769.02

*E, East; W, West*.

**Figure 4:**
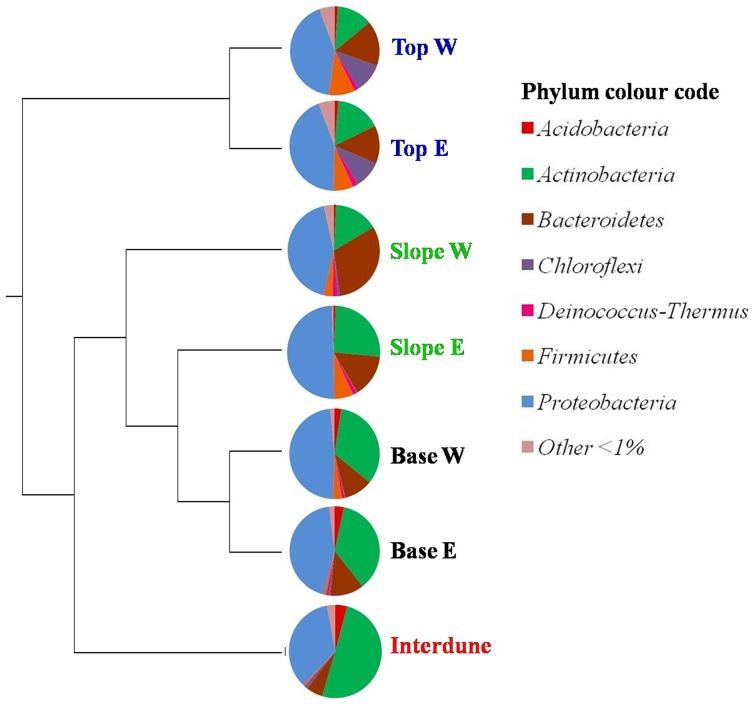
**UPGMA cluster analysis comparing the bacterial community composition in the seven ***a priori*** defined zones of the Kahani dune system**. E, East; W, West. The Pie charts depict dune zone-specific bacterial community composition at the phylum level (90% similarity cut-off).

After resampling this dataset for sequence consistency (*n* = 4653 reads), collector's curves using the Chao1 index indicated that dune Tops (E+W) and Slopes (E+W) samples reached near saturation (Figure S2). UPGMA analysis shows that bacterial communities cluster strongly in relation to their dune biotope of origin (Figure 4), supporting the T-RFLP analysis. However, while bacterial T-RFLP fingerprinting did not differentiate the east/west dune slopes (Figure 3), pyrosequencing analysis detected minor differences (Figure 4) which could be artifactual and related to the low number of samples analyzed.

**Figure 5:**
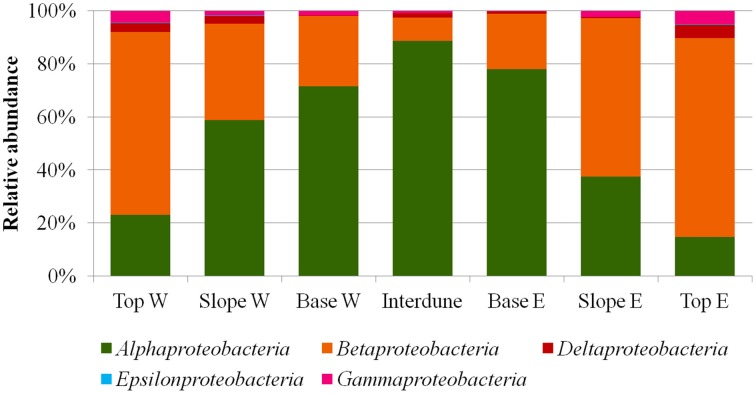
**Relative abundances (%) of the different proteobacterial classes in the seven ***apriori*** defined zones of the Kahani dune system**.

A total of thirty bacterial phyla were observed in the dune samples (Table S1), with only seven (*Acidobacteria, Actinobacteria, Bacteroidetes, Chloroflexi, Deinococcus*/*Thermus, Firmicutes*, and *Proteobacteria*) showing relative abundances greater than 1% (Figure 4). *Proteobacteria, Actinobacteria*, and *Bacteroidetes* were the dominant phyla in the 7 dune environments studied (Table S1). Bacterial phyla habitat-filtering could be observed with *Proteobacteria* dominating sand dune samples (i.e., Top, Slope and Base), ranging from 42 to 49%, and *Actinobacteria* dominating the Interdune biotope (51%; Figure 4). Moreover, the *Chloroflexi, Firmicutes*, and *Acidobacteria* (Figure 4) as well as the *Alpha*-, *Beta*-, *Gamma*-, and *Deltaproteobacteria* (Figure 5) displayed dune habitat-specific relative abundances: *Chloroflexi, Firmicutes, Alpha*-, *Gamma*-, and *Delta*-*proteobacteria* presented decreasing abundances from dune Top to the Interdune, while *Acidobacteria* and *Betaproteobacteria* presented the opposite trend (Figure 4). Similarly, the three most abundant genera of the dominant *Actinobacteria* (*Geodermatophilus* sp., *Blastococcus* sp., and *Arthrobacter* sp.) and *Proteobacteria* (*Microvirga* sp., *Massilia* sp., and *Novosphingobium* sp.) phyla displayed dune habitat specific abundances (Table S2). Altogether, these results strongly suggest habitat-filtration to be a determining process defining dune bacterial community assemblies.

This is confirmed by the fact that, apart from the dune Tops, each dune zone presented different and significantly associated (*p* < 0.05) bacterial indicator species (Dufrêne and Legendre, 1997). Two actinobacterial OTUs were associated with the Interdune: OTU24 from the genus *Geodermatophilus* sp. and OTU88, a *Modestobacter* sp. Three actinobacterial genera were also significantly linked to the eastern dune Slope (*Kineococcus* sp. [OTU73 and OTU77], *Arthrobacter* sp. [0TU16] and *Rathayibacter* sp. [0TU46]). Contrastingly, the two west dune slope indicator species were from the *Flavisolibacter* sp. (OTU6) and *Microvirga* sp. (OTU23) genera. Finally the dune Bases (east and west) were characterized by indicator species OTU74 from the Acidobacteria Gp16 class and OTU85 from the genus *Kineococcus* sp.

### Dune zone-specific metabolizing bacterial community

**Figure 6:**
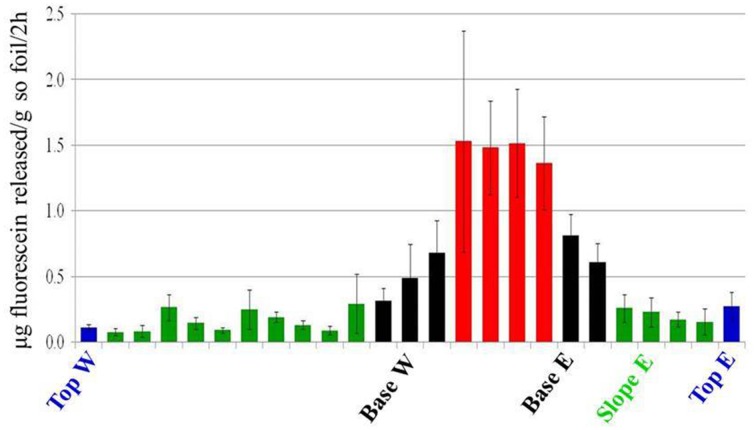
**Fluorescein di-acetate (FDA) hydrolysis values showing the average potential bacterial activity (μg fluorescein g^−1^ soil h^−1^) along the dune/interdune transect**. Each column of the histogram shows the mean and standard deviation of the values from the 5 parallel samples of each transect. The transect profile is represented from west (W) to east (E). Blue, Top; Green, Slope; Black, Base; Red, Interdune.

FDA hydrolysis was used as a proxy for the active bacterial metabolism (Chrzanowski et al., 1984). Active bacterial communities were detected in all dune areas studied (Figure 6), with the highest activity detected in the Interdune samples (average values 0.7–0.75 μg fluorescein g^−1^ soil h^−1^). Lowest activity was observed in Top and Slope samples (0.05–0.15 μg fluorescein g^−1^ soil h^−1^) with Base samples showing intermediate values (0.15–0.4 μg fluorescein g^−1^ soil h^−1^). The results of the Tukey's HSD test on the FDA hydrolysis data indicated that there were only three statistically distinct functional bacterial communities in the dune system: Interdune, Base and Slope/Top (Figure 6).

### Identification of environmental factors shaping dune bacterial community structures

**Figure 7:**
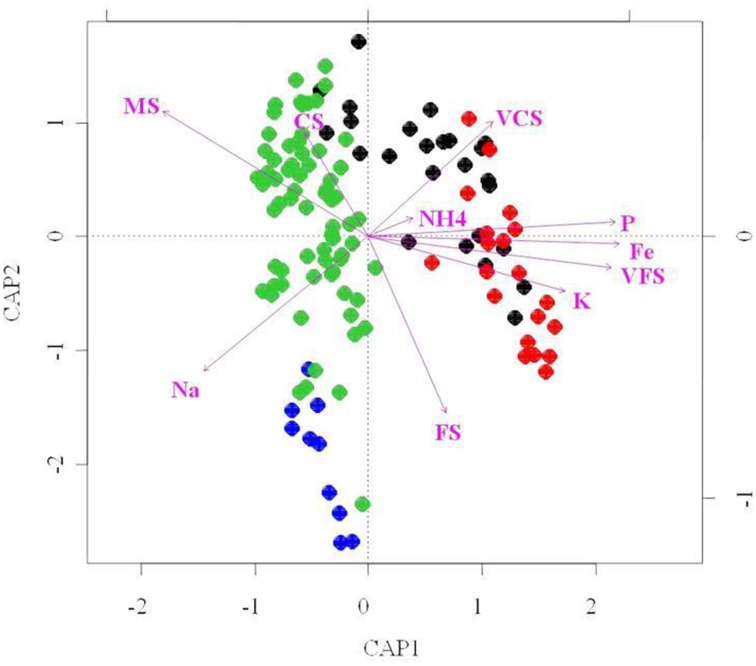
**Redundancy analysis (RDA) bi-plot of bacterial diversity and physico-chemical parameters**. Only the environmental variables that significantly explained variability in microbial community structures are fitted to the ordination (arrows). The direction of the arrows indicates the direction of maximum change of that variable, whereas the length of the arrow is proportional to the rate of change. Blue, dune Top; Green, dune Slope; Black, dune Base; Red, Interdune.

To further investigate relationships between abiotic factors and bacterial community structure (i.e., T-RFLP results), we performed redundancy analysis (RDA; Figure 7). RDA indicated that of the 21 soil attributes measured, 15 parameters were significant in explaining bacterial community structure variability (*p* < 0.01). However, taken together they only explain 21% of this variability. These included mineral [Na, K, P, NH_4_], metallic [Fe, Mn, Al] and granulometry factors [VCS, CS, MS, FS and (VFS/SC/ST/CY)], and nutrients [P, NH_4_]. Particle size distribution of the sand dune soils was an important environmental determinant to emerge from the RDA analysis. Our data (Table 1) show very clear grain size segregation, with the finest sands, silts and clays predominantly in the Interdune samples and the medium and coarser grained sands on the dunes themselves. The data indicated that fine grains, silts and clays [VFS, SC, ST, CY] are positively correlated with metals [Fe/Mn/Al], minerals [Ca/Mg] and some nutrients (C, P, NO^−^_3_-N, NH^+^_4_-N) (Figure 7 and Figure S1C).

Two main clusters of bacterial community samples were separated along the RDA axis1 as shown on Figure 7: the dune Base and Interdune bacterial community samples (cluster 1) and the dune Slope and Top samples (cluster 2). The bacterial community structures from cluster 1 were mainly driven by VFS, VCS and NH^+^_4_-N, K, Fe, and P contents while those of cluster 2 by MS and Na contents. Communities from both clusters were further separated along the RDA axis2 according to their dune biotope of origin, with granulometry parameters [FS and CS] differentiating the structures of the Interdune and dune Base communities as well as the ones from the dune Top and Slope (Figure 7).

Overall, these results demonstrate that environmental gradients in the four defined biotopes (i.e., dune Top, Slope, Base, and Interdune) significantly impact the bacterial community structures of dune/interdune transects.

## Discussion

The geomorphology and the macro-fauna/flora of the 34,000 km^2^ area of the Namib Sand Sea have been extensively studied (Seely and Pallett, 2008; Livingstone, 2013). However, arguably the most productive component of this depauperate environment, the edaphic microbial community (Makhalanyane et al., 2015), has received little attention at either local (a unique dune system) or regional (multiple dune systems) scales (Jacobson, 1997). Moreover, the drivers of desert dune microbial community assembly generally remain unclear.

This study was designed to evaluate the importance of deterministic processes (e.g., habitat filtering) in influencing bacterial communities in the Namib Sand Sea dune system. We provide a comprehensive analysis of a localized site, with 125 soil samples collected from five parallel 2 km dune/interdune transects. All samples were characterized by 16S rRNA gene T-RFLP fingerprinting and the determination of multiple physico-chemical parameters. The use of multiple parallel transects was intended to minimize the effects of local heterogeneity and spatial unevenness, and to generate statistically robust and representative datasets (Prosser, 2010). 16S rRNA gene amplicons from representative samples were also pyrosequenced to provide more insight into the phylogenetic composition of dune/interdune transects and evaluate if *a priori* defined niches would present different community assemblages.

### Soil properties of the Namib desert dune/interdune transect define four biotopes

The physico-chemical characteristics of soils along the Namib Desert dune-interdune transects clustered into four distinct datasets (Figure 2; Table 1): dune Top, Slope, Base, and Interdune. Granulometry data were typical for Namib Desert dune systems, with the highest proportion of fine sand on dune summit; a phenomenon commonly attributed to segregation of aeolian deposits from the gravel Interdune zone, and general concentration of coarse sands toward the dune Base (Lancaster, 1981). High Na content in the dune top samples is thought to be the result of salt capture from marine fog, the commonest source of moisture in this particular Namib Desert environment (Abrams et al., 1997; Hachfeld, 2000; Eckardt et al., 2013b). The high metal content recorded in the Interdune zone is likely to reflect the underlying geology, which includes red Tsondab sandstone (Besler, 1996), but may also be a consequence of the higher proportion of the fine soil particle fractions (VFS, SC, ST, and CY), the presence of which reduce percolation and enhance mineral accumulation and persistence (Chamizo et al., 2012).

### Bacterial community structures, diversities and potential function suggest habitat filtration in dune/interdune transects

A total of 30 bacterial phyla were detected in Namib Desert dune soils and all samples were dominated by *Proteobacteria, Actinobacteria*, and *Bacteroidetes*, which has also been observed in other desert systems (Makhalanyane et al., 2015). Namib Desert dune samples showed similar phylogenetic profiles to dune systems from the Gobi (Mongolia) and Taklamaken (China) deserts (An et al., 2013), but with lower OTU diversity than dune slope and interdune of the Gurbantunggut (China) desert (Li et al., 2015). The latter, however, was characterized by cyanobacteria-rich biological soil crusts (BSC), which were not observed in the Namib Sand Sea at the time of sampling.

Both 16S rRNA gene T-RFLP (Figures 3, 7; Figure S1A) and pyrosequencing (Figures 4, 5; Table 1 and Table S1; Figure S1B) data strongly support the concept of niche-partitioning/habitat-filtering in the Namib Desert dune/interdune system (Dumbrell et al., 2010), as both the structures and phylogenetic compositions of the bacterial communities demonstrated similar clustering than those of the physico-chemical characteristics (Figure 2). This was further supported by the low number of OTUs (15% from T-RFLP and 2% from pyrosequencing) shared by all dune biotopes (Figure S1). Taken together, these results strongly imply that habitat filtering, possibly *via* environmental physico-chemical gradients, is a major driver of bacterial community composition in dune ecosystems (Brankatschk et al., 2013; Liu et al., 2014).

While bacterial community T-RFLP fingerprinting and pyrosequencing showed highly comparable overall microbial community patterns (Figures 3, 4), pyrosequencing data showed minor differences in bacterial community structures between the east and west dune slopes (Figure 4). As the edaphic characteristics of these dune aspects were not significantly different, it is suggested that other environmental parameters, such as aeolian disturbance (Thomas and Dougill, 2007) or solar radiation (Jacobs and Sundin, 2001) differentials, could account for these differences.

### Dune/interdune transect habitat-filters

Distance based RDA analysis showed that 15 edaphic parameters, including mineral [Na, K], metallic [Fe/Mn/Al] and granulometry factors [VCS, CS, MS, FS and (VFS/SC/ST/CY)], and nutrients [P, NH_4_] were strongly correlated with bacterial community structure variability in dune soil environments (Figure 7, Table 1). Using Na as a proxy for soil salinity, we conclude that this factor plays an important role in directing dune microbial community compositions. Variations in salinity have previously been shown to be a strong bacterial habitat filter in Namib Desert gravel plain soils and hypoliths (Stomeo et al., 2013). The importance of soluble salts in dictating bacterial species abundance is supported by the observation that halotolerant microorganisms are readily isolated from interdune soils in the Negev Desert (Yu and Steinberger, 2012b) and Thar Desert sands (Sharma et al., 2013).

The role of K as a habitat filter may be related to intra-cellular osmoregulation (Fierer et al., 2012) while that of metals [Fe/Mn/Al] could be linked to their bioavailability, typically controlled by solubility and/or adsorption onto mineral surfaces (Cornell and Schwertmann, 2003). In this resource-limited desert environment, it was unsurprising to identify the nutrients P and NH_4_ as environmental drivers of dune bacterial community structures. The bioavailability of phosphorus and nitrogen has indeed previously been found to impact desert soil microbial functional and structural properties (Austin et al., 2004; Bell et al., 2014). We note that the use of P-Bray I to quantify total P underestimated the P contents in these mildly alkaline soils (Bowman and Vigil, 2002). In future studies, a more appropriate method such as the sodium bicarbonate procedure (Olsen et al., 1954) should be used to properly evaluate the bioavailable P contents in these desert soils and its potential as a factor shaping dune bacterial communities. The precise role of these dbRDA-determined environmental parameters in shaping dune bacterial community structures and functions could be further tested in controlled experiments (e.g., micro- or mesocosms; Lan et al., 2014).

The single most important environmental determinant to emerge from these data is the desert dune soil particle size distribution. Specific microbe-particle associations have already been demonstrated (e.g., Zhang et al., 2007; Carson et al., 2010). This could be related to the fact that differences in soil structure can manipulate soil nutrient status (Zhang et al., 2007; Carson et al., 2010). Our data do indicate that fine grains, silts and clays [VFS, SC, ST, CY] are positively correlated with metals [Fe/Mn/Al], minerals [Ca/Mg] and some nutrients (C, P, NO^−^_3_-N, NH^+^_4_-N) (Figure 2). This suggests that the relationship between bacterial diversity, soil structure and nutrient contents needs further investigation in dune soils as subtle differences in sand mineralogy and micronutrient contents has been shown to significantly impact edaphic community structuring (Welz et al., 2014).

We noted that 79% of the variation in soil bacterial community structures remains unexplained (Figure 5), although this is not uncommon in microbial ecology studies (e.g., Cottenie, 2005; Dumbrell et al., 2010). The high proportion of unaccounted variation might be attributed to other deterministic environmental factors not recorded in this study (e.g., wind transport, effect of sand burial stress or changes in humidity, and/or other soil physicochemistries) or the effects of microbial interactions (Vellend, 2010; Caruso et al., 2011). We also cannot rule out the role of stochastic processes in the assembly of dune communities, as, at a global scale, it has been shown significantly drive bacterial community assembly in deserts (Caruso et al., 2011).

The Namib Desert sand dunes are physically dynamic environments with high levels of wind erosion and dispersion (Eckardt et al., 2013a; Livingstone, 2013). It could be argued that in apparently well-connected habitats such as those of the Namib Sand Sea, dispersal rates should be sufficiently high so as to lead to homogenization of microbial communities due to mass-mixing effects (Leibold and Norberg, 2004). However, our bacterial fingerprint results and phylogenetic analyses show that only a limited number of OTUs were shared across all four dune habitats and that the number of OTUs unique to each area and OTUs shared by contiguous habitats was also low (Figure S1). This suggests that aeolian transport and cellular dispersal is not a substantial driver of sand dune bacterial community structure. However, it cannot be completely excluded. Indeed, in the dune Top zone, which experiences the highest wind disturbance (Eckardt et al., 2013a), the lowest levels of bacterial activity were observed (as determined by fluorescein diacetate hydrolysis: Figure 6) and no indicator species was identified. It could be argued that this zone does not represent a stable community and that the detected activity could be derived from microorganisms bound to wind-transported mineral grains (Yamaguchi et al., 2012).

### Phylogenetic composition of Namib desert dune biotope soils in relation to pedogenesis and ecosystem development

Many studies have shown that microbial communities evolve with the development of ecosystems, over short (weeks) or long (years/hundreds of years) temporal scales (e.g., Ramond et al., 2012; Ferrenberg et al., 2013; Jangid et al., 2013). For example, in a 6500 year dune soil chronosequence, *Betaproteobacteria, Bacteroidetes, Actinobacteria, Cyanobacteria*, and *Firmicutes* were shown to dominate “young soil ecosystems”; i.e., recent or often-disturbed soil environments (Jangid et al., 2013). Contrastingly, the relative abundances of *Acidobacteria, Alphaproteobacteria*, and *Planctomycetes* were higher in “aged soils”; i.e., more structured and stabilized environments (Jangid et al., 2013). Similarly, continuous physical disturbance has been shown to influence community composition in cold desert biocrust soils (Kuske et al., 2012), with continuously trampled soils showing decreasing cyanobacterial abundances with corresponding increases in *Actinobacteria, Chloroflexi*, and *Bacteroidetes*, compared to undisturbed soils.

As previously observed in a 6500 year dune soil chronosequence (Jangid et al., 2013), *Betaproteobacteria, Bacteroidetes*, and *Firmicutes* were more abundant in the more dynamic dune Tops and Slopes, while the relative abundances of *Acidobacteria* and *Alphaproteobacteria* were higher in the more stable Interdune soils. *Cyanobacteria* were almost exclusively detected in the stable Interdune soils (albeit in proportionally very low percentages: 0.46%), which were dominated by *Actinobacteria*, comprising over 50% of the interdune community (Table S1). Our results are remarkably consistent with the comparative data obtained from disturbed and stable biocrust systems (Kuske et al., 2012) and suggest that the degree of stability or perturbation of the Namib Desert soils may be one of the key deterministic factors contributing to the high proportion of unaccounted variation in community structure of the Namib Desert dune systems.

## Concluding remarks

Our results clearly showed that, in dune/interdune transects of the Namib Desert, soil physico-chemistry define four dune zones, namely the dune Tops, Slopes, Bases and Interdune. Furthermore, we demonstrated that each of these zones presented unique bacterial communities. Altogether, this strongly suggested that these communities were selected through habitat filters, i.e., environmental deterministic factors (Dumbrell et al., 2010). The measurement of 21 environmental factors enabled us to define that habitat stability, soil texture and mineral and nutrient contents were influencing dune/interdune transect bacterial communities. However, almost 80% of the dune/interdune bacterial community's variability remained unexplained indicating that other unmeasured deterministic factors or processes, such as species' interaction, dispersal and/or stochasticity (Vellend, 2010), may be involved. Consequently, to further investigate bacterial community assembly and its driver(s) in dune systems, such study should be extended to multiple dune/interdune environments.

## Author contributions

SR, JR, and DC designed the experiment. SR performed the lab work and analyzed the data with some help from JR. JR, BJ, SR, MS, and DC participated in writing the manuscript. DC contributed to the reagents, materials and analysis tools required. MS' field knowledge was necessary in the design experiment.

### Conflict of interest statement

The authors declare that the research was conducted in the absence of any commercial or financial relationships that could be construed as a potential conflict of interest.
