# Supplementary material for: Namib Desert dune/interdune transects exhibit habitat-specific edaphic bacterial communities
Source: Front Microbiol. 2015 Sep 4;6:845. doi: 10.3389/fmicb.2015.00845 (PMC4560024; doi:10.3389/fmicb.2015.00845)
Supplement: Supplementary file 1 [file Table1.DOCX]

| Phylum | Top w | Slope W | Base W | Interdune | Base E | Slope E | Top E |
| --- | --- | --- | --- | --- | --- | --- | --- |
| *Proteobacteria* | 42.27 | 43.08 | 48.00 | 34.90 | 44.09 | 49.57 | 43.89 |
| *Actinobacteria* | 12.90 | 15.71 | 33.48 | 50.46 | 36.11 | 25.81 | 16.36 |
| *Bacteroidetes* | 16.41 | 31.33 | 10.24 | 5.76 | 12.19 | 14.95 | 13.65 |
| *Firmicutes* | 9.44 | 3.02 | 3.16 | 0.52 | 0.90 | 6.66 | 7.07 |
| *Chloroflexi* | 10.74 | 0.77 | 0.25 | 1.18 | 0.68 | 0.20 | 9.99 |
| *Acidobacteria* | 1.13 | 0.82 | 2.40 | 4.10 | 3.31 | 0.71 | 1.39 |
| *Deinococcus-Thermus* | 1.51 | 1.79 | 0.92 | 0.17 | 0.73 | 1.45 | 1.84 |
| *Armatimonadetes* | 0.82 | 0.68 | 0.53 | 0.62 | 0.68 | 0.17 | 1.41 |
| *Verrucomicrobia* | 1.34 | 0.87 | 0.19 | 0.30 | 0.12 | 0.07 | 1.03 |
| *Planctomycetes* | 1.25 | 0.33 | 0.10 | 0.24 | 0.14 | 0.01 | 1.23 |
| *Gemmatimonadetes* | 0.15 | 0.66 | 0.28 | 0.41 | 0.29 | 0.07 | 0.32 |
| *Aquificae* | 0.68 | 0.08 | 0 | 0.09 | 0.04 | 0.03 | 0.77 |
| *Acetothermia* | 0.56 | 0.11 | 0.01 | 0.13 | 0.14 | 0 | 0.65 |
| *Candidatus Saccharibacteria* | 0.03 | 0.30 | 0.15 | 0.13 | 0.31 | 0.18 | 0.03 |
| *Thermodesulfobacteria* | 0.09 | 0.06 | 0.09 | 0.28 | 0.18 | 0.03 | 0.13 |
| *Ignavibacteriae* | 0.25 | 0 | 0 | 0 | 0 | 0 | 0.07 |
| *Tenericutes* | 0.12 | 0.10 | 0.07 | 0 | 0 | 0.01 | 0.03 |
| *candidate division WPS-2* | 0.08 | 0.12 | 0.04 | 0 | 0.01 | 0 | 0.03 |
| *Cyanobacteria/Chloroplast* | 0 | 0 | 0 | 0.43 | 0 | 0.06 | 0 |
| *Nitrospirae* | 0.01 | 0.09 | 0.06 | 0.04 | 0.01 | 0.01 | 0.01 |
| *candidate division WPS-1* | 0.05 | 0.03 | 0 | 0.17 | 0.03 | 0 | 0 |
| *Deferribacteres* | 0.10 | 0 | 0 | 0 | 0 | 0 | 0 |
| *candidate division ZB3* | 0 | 0.04 | 0 | 0.04 | 0.04 | 0 | 0 |
| *Synergistetes* | 0.03 | 0 | 0 | 0 | 0 | 0 | 0.03 |
| *Lentisphaerae* | 0.03 | 0 | 0 | 0 | 0 | 0 | 0.03 |
| *Omnitrophica* | 0.01 | 0 | 0 | 0 | 0 | 0 | 0.03 |
| *Parcubacteria* | 0.02 | 0 | 0 | 0 | 0 | 0 | 0 |
| *Elusimicrobia* | 0 | 0 | 0 | 0.02 | 0 | 0 | 0 |
| *Hydrogenedentes* | 0 | 0 | 0.01 | 0 | 0 | 0 | 0 |
| *Thermotogae* | 0 | 0 | 0.01 | 0 | 0 | 0 | 0 |

**Supplementary Table S1.** Relative abundances (%) of the bacteria phyla detected by 16S rRNA gene pyrosequencing in the soils of the seven dune zones studied. E: East / W: West
