# Supplementary material for: Namib Desert dune/interdune transects exhibit habitat-specific edaphic bacterial communities
Source: Front Microbiol. 2015 Sep 4;6:845. doi: 10.3389/fmicb.2015.00845 (PMC4560024; doi:10.3389/fmicb.2015.00845)
Supplement: Supplementary file 2 [file Table2.DOCX]

| Phylum | Family | Genus | Top W | Slope W | Base W | Interdune | Base E | Slope E | Top E |
| --- | --- | --- | --- | --- | --- | --- | --- | --- | --- |
| *Actinobacteria* | *Geodermatophilaceae* | *Geodermatophilus* | 0.40 | 1.50 | 1.57 | 9.69 | 3.04 | 0.92 | 0.19 |
|  | *Geodermatophilaceae* | *Blastococcus* | 0.25 | 1.20 | 0.67 | 9.03 | 2.57 | 0.66 | 0.25 |
|  | *Micrococcaceae* | *Arthrobacter* | 0.50 | 1.63 | 7.18 | 3.29 | 9.95 | 8.55 | 0.14 |
| *Proteobacteria* | *Methylobacteriaceae* | *Microvirga* | 1.58 | 13.49 | 20.13 | 19.21 | 22.40 | 8.63 | 0.16 |
|  | *Oxalobacteraceae* | *Massilia* | 8.88 | 5.92 | 7.28 | 1.33 | 5.57 | 18.44 | 5.17 |
|  | *Sphingomonadaceae* | *Novosphingobium* | 7.76 | 1.63 | 1.42 | 0.41 | 0.77 | 1.85 | 2.32 |

**Supplementary Table S2. Relative abundances (%) of the three most abundant genera of the dominant colonist phyla from the interdune (*Actinobacteria*) and from the dune (*Proteobacteria*).** E: East / W: West.
