# Supplementary material for: Namib Desert dune/interdune transects exhibit habitat-specific edaphic bacterial communities
Source: Front Microbiol. 2015 Sep 4;6:845. doi: 10.3389/fmicb.2015.00845 (PMC4560024; doi:10.3389/fmicb.2015.00845)
Supplement: Supplementary file 3 [file Presentation1.PDF]

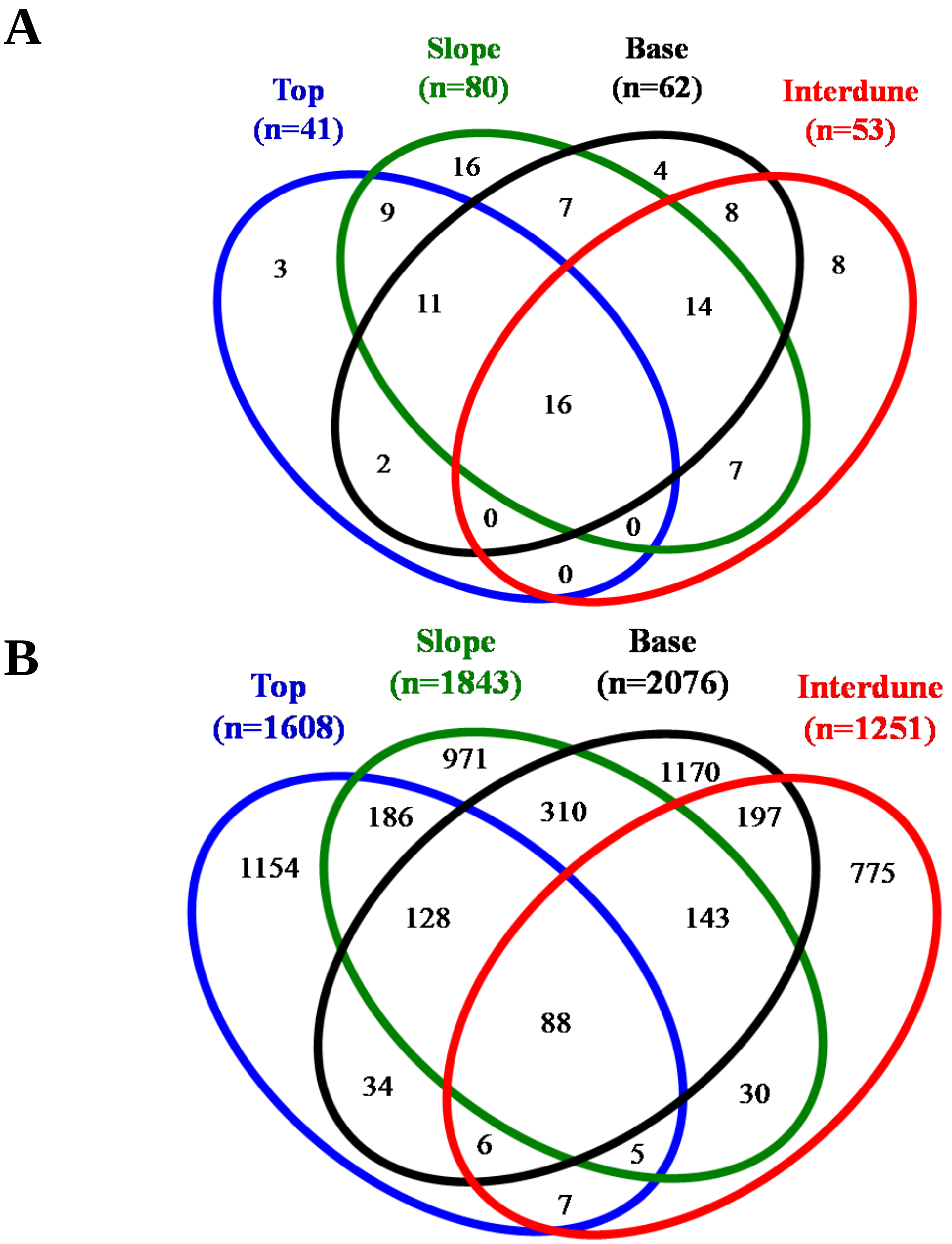

Supplementary Figure S1. Venn diagram depicting OTU distribution in the different dune biotopes. A. Distribution of T-RFs. **B.** Distribution of OTUs (97% similarity) obtained from pyrosequencing analysis. n = number of OTUs per zone.

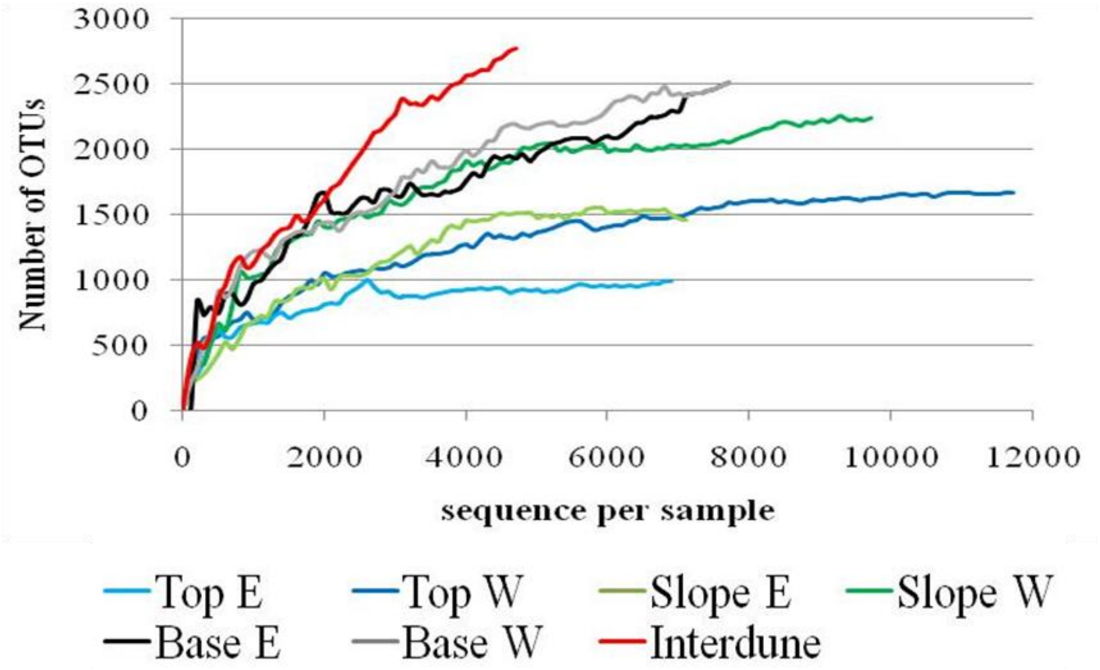

Supplementary Figure S2. Rarefaction curve (Chao1 index) generated from 16S rRNA gene 454 pyrosequencing using observed OTUs (97% similarity). E: East / W: West
